# Case Report: A Novel AChR Epsilon Variant Causing a Clinically Discordant Salbutamol Responsive Congenital Myasthenic Syndrome in Two Egyptian Siblings

**DOI:** 10.3389/fneur.2022.909715

**Published:** 2022-06-02

**Authors:** Marta Gómez-García de la Banda, Emmanuel Simental-Aldaba, Nagia Fahmy, Damien Sternberg, Patricia Blondy, Susana Quijano-Roy, Edoardo Malfatti

**Affiliations:** ^1^Pediatric Neurology and ICU Department, AP-HP Université Paris Saclay, Hôpital Raymond Poincaré, Garches, France; ^2^Reference Center for Neuromuscular Diseases Centre “Nord- Est- Ile de France”, FILNEMUS, Creteil, France; ^3^European Reference Center Network (Euro-NMD ERN), Paris, France; ^4^APHP, Centre de Référence de Pathologie Neuromusculaire Nord-Est-Ile-de-France, Henri Mondor University Hospital, Créteil, France; ^5^Department of Neurorehabilitation, Instituto Nacional de Rehabilitación “LGII”, Mexico City, Mexico; ^6^Neuromuscular Unit, Faculty of Medicine, Ain Shams University, Cairo, Egypt; ^7^Service de Biochimie Métabolique, Centre de Génétique, Groupe Hospitalier Pitié-Salpêtrière, APHP Sorbonne Université, Paris, France; ^8^Centre de Recherche en Myologie, UMRS974, Paris, France; ^9^Univ Paris Est Créteil, INSERM, IMRB, Créteil, France; ^10^AP-HP, Hôpital Mondor, Service d'histologie, Créteil, France

**Keywords:** congenital myasthenic syndrome, *CHRNE*, neuromuscular junction, β2 adrenergic agonists, salbutamol

## Abstract

Congenital myasthenic syndromes (CMS) are inherited disorders that lead to abnormal neuromuscular transmission. Post-synaptic mutations are the main cause of CMS, particularly mutations in *CHRNE*. We report a novel homozygous *CHRNE* pathogenic variant in two Egyptian siblings showing a CMS. Interestingly, they showed different degrees of extraocular and skeletal muscle involvement; both presented only a partial response to cholinesterase inhibitors, and rapidly and substantially ameliorated after the addition of oral β2 adrenergic agonists. Here, we enlarge the genetic spectrum of *CHRNE*-related congenital myasthenic syndromes and highlight the importance of a β2 adrenergic agonists treatment.

## Introduction

Congenital myasthenic syndromes (CMS) are a heterogeneous group of rare inherited neuromuscular transmission disorders characterized by fluctuant muscle weakness and fatigability, classically described as non-progressive disorder with a low impact on the walking ability (1). Their estimated prevalence is 1 in 500,000 in Europe (2), with higher frequency in some geographic areas with high consanguinity rates, such as North Africa (3). To date, mutations in more than 30 genes are known to be associated with CMS, which can be classified into pre-synaptic, synaptic, and post-synaptic, according to the location of the molecular defect (4, 5).

Post-synaptic defects have been reported to be the most frequent cause in more than half of CMS, particularly those due to mutations in the gene encoding the ε subunit of the acetylcholine receptor (*CHRNE*) (5, 6).

Mutations of *CHRNE* gene are associated with two types of mechanisms: a low expression of functional ACh receptors (AChR) at the cell surface, causing a primary acetylcholine receptor deficiency, also called “low expressor” CMS, or a kinetic defect in the acetylcholine receptor, where the amount of AChR is normal, but there is an alteration in the channel opening time after acetylcholine binding, leading either to slow channel syndrome, if the opening time is excessively prolonged, or to fast channel syndrome, if the closure is too fast (7).

The typical CMS presentation occurs in infancy with marked fatigability, ophthalmoparesis, bilateral ptosis, mildly progressive facial, bulbar, axial, and limb weakness with respiratory involvement related to myasthenic crisis (8, 9).

Clinical manifestations of AChR ε subunit deficiency may vary considerably between affected families (10), and the same family members. This results in a heterogeneous phenotype between siblings with different clinical courses and prognoses (11, 12).

The primary therapeutic intervention in low expressor *CHRNE*-associated CMS is the cholinesterase inhibitor pyridostigmine. Reported treatment experience with low expression *CHRNE* mutations shows that cholinesterase inhibitors cause a favorable but incomplete response, improving muscular strength, particularly in ocular muscles. In poorly or incompletely responsive patients, other therapies have been considered, such as beta2 adrenergic receptor agonists (albuterol, salbutamol) or the 3,4- diaminopyridine (21).

The use of 3,4- diaminopyridine results in further improvement when the response to initial therapy is not sufficient or incomplete (13). The addition of beta2 adrenergic receptor agonists stabilizes the neuromuscular junction structure and reduces the acetylcholine dispersion, thus producing a dramatic improvement in some cases (14). This is different from other CMS, as, for instance, slow channel CMS or *DOK7* CMS, where the addition of pyridostigmine potentially worsens symptoms (15).

Here, we report on two siblings with a clinically heterogeneous CMS linked to a novel *CHRNE* variant and describe their variable therapeutic response to pyridostigmine, and oral β2 adrenergic agonists.

## Case Report

Patient 1 (P1), an 11-year-old boy, and Patient 2 (P2), an 8-year-old girl, are the third and fourth born of a sibship of four, born to healthy consanguineous Egyptian parents. The first-born deceased at 5 years, following a respiratory infection, suggesting a probable neuromuscular involvement.

P1 presented at 8 months with ptosis, ophthalmoplegia, poor cry, and nasal regurgitations. His psychomotor development was normal, but he developed fatigable generalized muscle weakness with frequent respiratory infections. Detection of antibodies against AChR and MuSK was negative; electrophysiologic studies showed a decremental response in the compound muscle action potential in response to repetitive stimulation in the median nerve. Serum lactate and CK levels were normal. On the base of clinical suspicion of a seronegative myasthenia gravis, a treatment with pyridostigmine was started at 6 months. The latter only showed a slight amelioration of the weakness and fatigability. P1 successively underwent plasma exchanges, repeated intravenous immunoglobulin therapy, and a thymectomy. The course was progressive and led to gait loss at 6 years.

**Figure 1:**
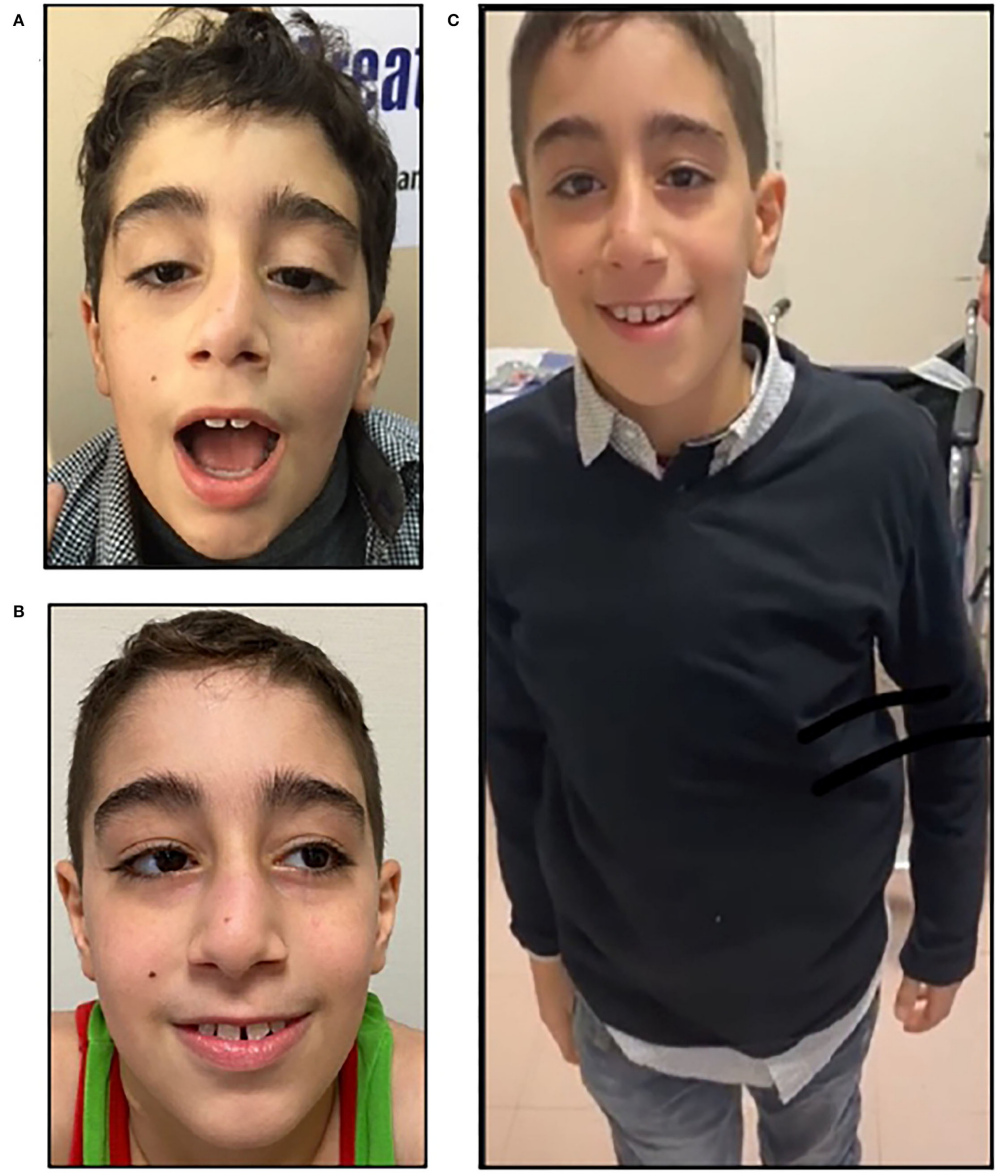
Clinical features, P1. P1, an 11-year-old boy with before treatment with salbutamol, showing severe bilateral ptosis and facial weakness **(A)**; note the improvement after salbutamol treatment; he showed only mild ptosis **(B)** and less fatigability, being able to walk without aid **(C)**.

Referred to our center at 8 years, he showed bilateral ptosis and ophthalmoplegia, nasal voice, facial weakness with open mouth, and diffuse muscle weakness, particularly in triceps and hamstrings (3 MRC), but also in deltoid, quadriceps, and psoas muscles (4 MRC). The *Garches* myasthenic score was 60/100, and he had a very restricted indoor ambulation, necessitating a wheelchair for the exteriors (Figure 1). Spirometry revealed respiratory involvement with a forced vital capacity (FVC) of 1,640 ml (89%) when sitting and 1,060 ml (59%) in supine position, highly suggesting a diaphragmatic weakness. EKG and cardiac ultrasonography were normal.

Patient 2 was born at term after an uneventful pregnancy and reached the normal milestones at expected age. She presented ptosis and frequent respiratory infections at 2 years old. ENMG showed a significant decremental response in repetitive nerve stimulation. There were no anti-AChR and anti-MUSK antibodies in serum. A therapy with Pyridostigmine 60 mg two times a day was started, and the response was partial, with fluctuating muscle weakness and fatigability despite adequate dosing (7 mg/ kg/day).

**Figure 2:**
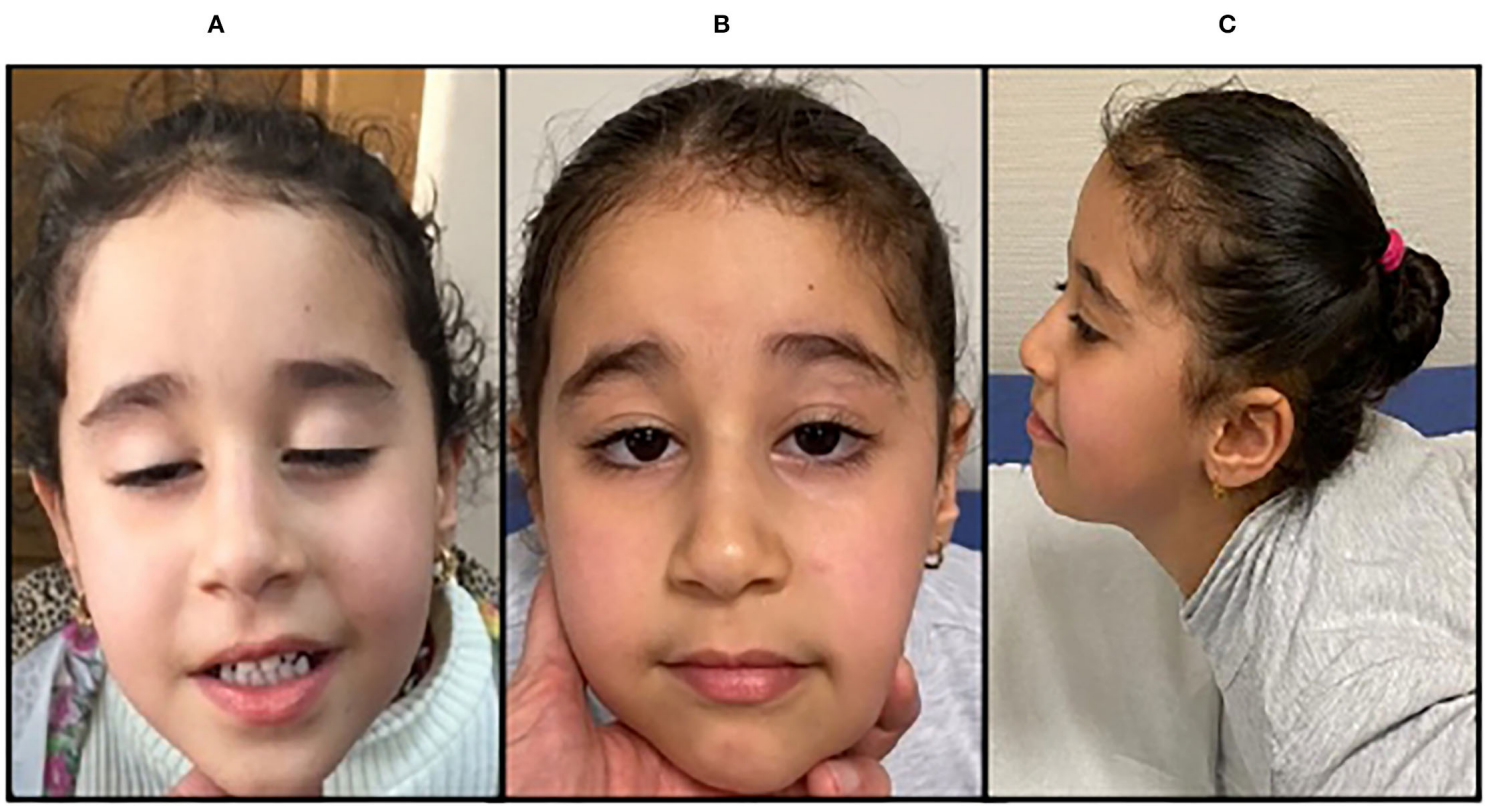
Clinical features, P2. P2, an 8-year-old girl presenting with sever bilateral ptosis before treatment with salbutamol **(A)**; amelioration of the eyelid ptosis **(B)**; amelioration of strength in neck extensor muscles **(C)**.

On referral to our clinic at 5 years old, neurological examination revealed symmetrical axial and proximal muscular weakness, bilateral ptosis, and facial weakness with open mouth (Figure 2). She had fluctuant fatigability, being able to walk with mild restrictions for 500 meters. 6MWT corresponded to 448 meters, and she scored 70/100 at *Garches'* myasthenic score. There was no bulbar involvement, including dysphagia and dysphonia. Respiratory test revealed an FCV of 133 ml (84%) in sitting positions and 1,900 ml (70%) in supine position. Cardiac workup was normal.

Based on the familial clinical story and the ancillary tests, a diagnosis of CMS was suspected in both children.

### Molecular Studies

Next-Generation-Sequencing (NGS)-based screening of 54 genes involved in muscular excitability (a list on request), among which 30 CMS causing genes, including *CHRNE*, was performed in the proband P1 using a SeqCapEZ capture design (Nimblegen), and a MiSeq sequencer (Illumina). A total of 1,101 coding sequences and flanking regions were sequenced. Variants were identified through a bioinformatics pipeline (Genodiag, Paris, France). Copy number variations (CNVs) in targeted regions were searched for by a dedicated algorithm based on comparison of normalized number of reads of each region among the 12 samples of the sequence run. Variants were filtered according to their frequency in the general population (GnomAD) and in the patient's samples. It revealed the presence of a homozygousc.632_633dupCGp. (Gly212Argfs^*^3) variant in the exon 7 of *CHRNE*gene. The same variant was detected by Sanger sequencing of *CHRNE* exon 7 in a homozygous state in Patient P2, and in a heterozygous state in both healthy parents.

**Figure 3:**
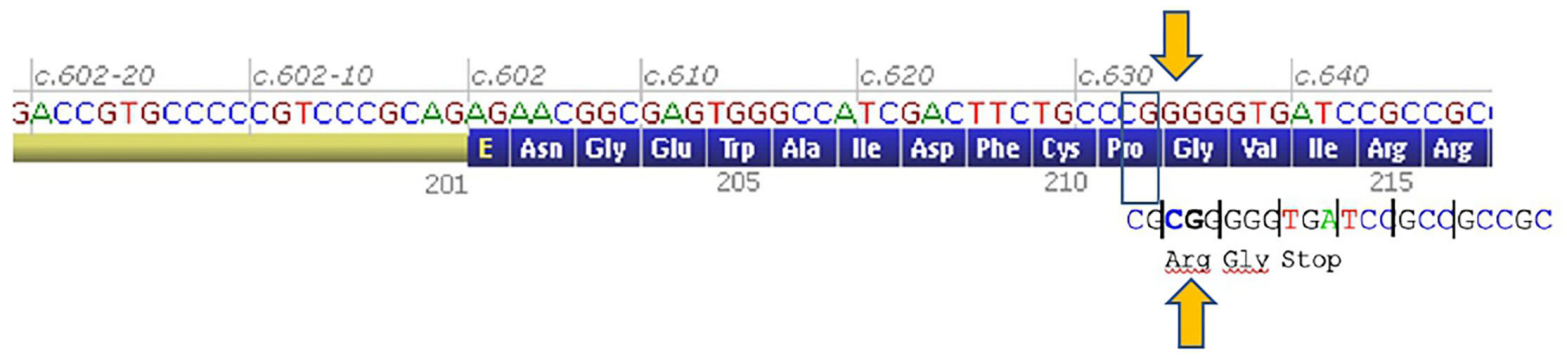
Genetics. A 2-bp shift of a reading frame from Codon 212 and premature stop codon at codon 214 of *CHRNE* gene (an image of this part of mRNA was taken from Alamut Visual software, Interactive Biosoftware Sophia Genetics).

This variant predicts a shift in a translation reading frame, a change of glycine 212 to arginine, and a premature stop codon at Codon 214 (Figure 3). This premature termination codon may trigger nonsense-mediated decay of *CHRNE* mRNAs. Such a homozygous frame shift in the *CHRNE* gene results in low expressor-type CMS.

### Response to Treatment

The clinical status before and after the introduction of Salbutamol is listed in Table 1. Both patients were initially treated with pyridostigmine from ages 6 months (P1) and 10 months (P2), with a favorable but incomplete response, despite adequate dosing (at least 1 mg/Kg/dose for at least 4 doses per day). We introduced a concomitant treatment with salbutamol, which was well tolerated without side effects, with a progressive dose until 6 mg/day in both children, (P1 at.17 mg/Kg/day; P2 at.2 mg/kg/day), taken in three divided doses. An important improvement of the weakness was observed in both P1 and P2 from the first 2 weeks of treatment, particularly in P1. At the 6-month visit, P1 was able to walk and run, using the wheelchair only for long distances, with a 6MWT of 390 mts. Ptosis and ophthalmoplegia were also partially ameliorated, with a 71/100 in the Garches Myasthenic score, corresponding to a 10-point amelioration. Importantly, the spirometry showed an FVC of 2,530 ml (94%) in sitting position, and 2,560 ml (95%) in supine position, highlighting a spectacular amelioration compared to the first assessment, 1,060 ml, 59% in supine position (Table 1).

**Table 1:** Response to treatment, adding oral β2 adrenergic agonists (salbutamol).

	**Before treatment with salbutamol**		**After treatment with salbutamol**	
	**P1**	**P2**	**P1**	**P2**
Facial involvement	Severe bilateral ptosis and ophthalmoplegia.	Bilateral ptosis and ophthalmoplegia.	Mild bilateral ptosis.	Mild ophthalmoplegia.
Muscle weakness	Global muscle weakness.	Proximal and axial muscular weakness.	Mild muscle weakness.	Normal
Walking ability	Very restricted ambulation.	Able to walk with mild restrictions.	Able to walk.	No restrictions to walk.
Myasthenic score (pts)	60/100	70/100	71/100	85/100
6MWT (mts)	Not able to walk more than 6 steps.	448	390	486
Diaphragm involvement	Present.	Absent.	Absent.	Absent.
FVC Sitting (ml)	1,640 (89%)	1,330 (84%)	2,530 (94%)	1,640 (103%)
FVC Supine (ml)	1,060 (59%)	1,900 (70%)	2,560 ml (95%)	1,460 (92%)

*Yo, years old; mo, months old; FVC, forced vital capacity*.

On the long term, P1 showed mild fluctuating symptoms, including increased fatigability and restricted ambulation some months after starting the treatment, and he showed a myasthenic crisis with extreme fatigue, loss of ambulation, swallowing difficulties, and diaphragmatic involvement (FVC of 36%) needing hospitalization. The fatigability and respiratory involvement disappeared in 1 day.

P2 with a milder clinical phenotype at the baseline significantly improved her axial strength and her ptosis after treatment, showing an amelioration of 15 points in the myasthenic score. 6MWT was 486, consisting of a gain of 38 meters. CV remained stable and without respiratory infections.

In these times, both patients follow treatment without any respiratory involvement, and mild-to-moderate motors symptoms with occasional fluctuations in time.

## Discussion

CMS are a heterogeneous group of inherited disorders caused by mutations in genes that lead to impaired neuromuscular transmission (15). Most CMS are caused by molecular defects in the acetylcholine nicotinic receptor of the muscle, in particular *CHRNE*, which accounts almost 80% of all CMS (5, 16, 17), leading to AChR deficiency (15).

Based on a similar clinical phenotype, consisting with poorly responding myasthenia and their family history, we suspected a congenital myasthenic syndrome in both P1 and P2. Genetic studies disclosed a novel never-reported variant mutation in *CHRNE*, confirmed by segregation studies. Frame shift mutations of *CHRNE* gene are associated with a low expression of the ACh receptors at the cell surface, termed as a “low expressor” CMS mechanism, which is the most common type of CMS.

Clinically, *CHRNE* have been described as mild and non-progressive disorders, but some authors have recently reported loss of ambulation in several patients (10, 11, 18–20). A wide spectrum symptom, even among individuals within the same family, has been reported, spanning from severe cases with premature and fatal respiratory failure and milder cases presenting only with ptosis (8, 11). P1 and P2 manifested a typical phenotype with early-onset bilateral ptosis, ophtalmoparesis, facial, neck muscle and proximal limb weakness, with fluctuant fatigability, discordant severity, but also a marked respiratory dysfunction and symptoms, generating a risk of life-threatening complications. Of note, the genetically unconfirmed first born of the sibship died of respiratory infection during infancy.

The precise mechanisms of this clinical variability discordance in severity are not well understood; they might be associated with epigenetic, varying-phenotype mutations, sex variation, and environmental factors (11). In any case, we stress the importance to monitor tightly the respiratory function in patients with ACHRε deficiency.

From a therapeutic standpoint, we only observed a partial response to esterase inhibitors as reported previously (13). The introduction of salbutamol resulted in more consistent response (Table 1), without side effects in both children.

The mechanism of action β2 adrenergic receptors agonist on the neuromuscular junction has remained unknown until today, but some studies revealed that they increase quantal release and reduce the conductance of the AChR channel (9) and increase the number of briefs intraburst closures, causing an open-channel blockade of AChR (21).

Treatment with salbutamol led to a clear respiratory benefit in both children, suggesting an improvement of diaphragmatic weakness. The association of the acetylcholinesterase inhibitors may lead to a significant impact, not just on their strength and fatigability, but also on their autonomy for daily life, without significant side effects, thus reinforcing that β2-adrenergic receptor agonist therapy could be the first choice of pharmacological strategy for treating CMS with *CHRNE* mutations, as previously reported (22).

Our observations and the review of the literature suggest that mutations in the genes coding for subunits of the acetylcholine receptor, and, in particular, the epsilon subunit, should be the first researched in patients presenting with symptoms of myasthenia and decremental response on repetitive stimulation in absence of anti-AchR and anti-musk antibodies. Mutations in the *CHRNE* with a low expressor profile may be identified in severe infantile cases and may have a mild response on acetylcholinesterase inhibitors. In these cases, adding β2 adrenergic agonists could, as in our cases, result in an important gain of motor and respiratory function (9, 23–25).

## Conclusion

In conclusion, these cases broaden the genetic spectrum of *CHRNE*-related myasthenic syndromes. Further studies are needed to confirm the relationship between this mutation, the progressive course, and the response to treatment.

## Data Availability Statement

The datasets presented in this article are not readily available because of ethical and privacy restrictions. Requests to access the datasets should be directed to the corresponding author.

## Ethics Statement

The studies involving human participants were reviewed and approved by Comité de Protection des Personnes Est IV DC-2012-1693. Written informed consent to participate in this study was provided by the participants' legal guardian/next of kin. Written informed consent was obtained from the individual(s), and minor(s)' legal guardian/next of kin, for the publication of any potentially identifiable images or data included in this article.

## Author Contributions

MG-G, NF, SQ-R, and EM: study conception, design, and supervision of the course of the project. MG-G, ES-A, NF, and PB: data collection, taking responsibility of patient follow-up, data management, and reporting. MG-G, ES-A, PB, DS, SQ-R, and EM: analysis and interpretation of results, taking responsibility of logical interpretation based on a literature review, and presentation of the results. MG-G, ES-A, DS, SQ-R, and EM: draft manuscript preparation and taking responsibility of the construction of the whole or body of the manuscript. All the authors reviewed the results, provided critical feedback, and approved the final version of the manuscript.

## Conflict of Interest

The authors declare that the research was conducted in the absence of any commercial or financial relationships that could be construed as a potential conflict of interest.

## Publisher's Note

All claims expressed in this article are solely those of the authors and do not necessarily represent those of their affiliated organizations, or those of the publisher, the editors and the reviewers. Any product that may be evaluated in this article, or claim that may be made by its manufacturer, is not guaranteed or endorsed by the publisher.
